# Chromosome Scale Assembly of Novel *Caenorhabditis* species #65 (JU4118)

**DOI:** 10.21203/rs.3.rs-9774361/v1

**Published:** 2026-05-22

**Authors:** Jasbelle Sosa, Michelle A. McCauley, Victoria K. Eggers, Janna L. Fierst, Karolina Willicott

**Affiliations:** Florida International University; Florida International University; Florida International University; Florida International University; Florida International University

## Abstract

We present the chromosome level assembly of strain JU4118, an inbred line of *Caenorhabditis* species #65. This species is a wild isolate that was sampled in Da Lat, Vietnam by Marie-Anne Félix’s lab in December 2019. This strain has a genome size of 126.4Mb, with a GC content of 37.96%. By assembling and annotating its genome, we aim to expand future evolutionary studies on the *Caenorhabditis* genus.

## Description

*Caenorhabditis* is a genus of the phylum of Nematoda that consists of diverse roundworms, including the species *Caenorhabditis elegans*. *C. elegans* has become an important model organism in biology. In an effort to understand further evolutionary biology of *Caenorhabditis*, many species have been sampled around the world to be sequenced and described [28]. Here, we aim to describe *Caenorhabditis* species #65 (strain JU4118), an outcrossing species. *Caenorhabditis* sp. 65 was isolated from rotting fruit in Da Lat, Vietnam by Marie-Anne Félix lab and was inbred for 25 generations.

Using PacBio HiFi long-read sequencing and Hi-C chromosome scaffolding and assembly data, we describe the chromosome-level genome assembly and annotation of an inbred strain of *Caenorhabditis* sp. 65, JU4118. With this reference genome, we provide a resource for future phylogenomic and evolutionary studies.

## Methods

Worms were kept in cryopreservation until transfer to our research group and were maintained continuously thereafter using standard *Caenorhabditis elegans* techniques. Worms were grown on agar plates at 20°C using nematode growth media and seeded with a lawn of OP50 strain *E. coli*. For DNA extractions, worm populations were expanded by transferring a small “chunk” of agar to three 100mm plates seeded with *E. coli* and left at 20°C for 2–3 days until plate was filled with mixed-age worms but free of dauer larvae. Worms were then washed off plates with M9 buffer into a 15mL conical tube and washed twice with M9 buffer to minimize surface contaminants, then resuspended in 10mL M9 and left on a rocker overnight (~ 17 hours) to purge gut of further contaminants. Prior to extraction, worms were washed twice more with M9. Worms were collected by pelleting worm bodies via centrifugation, removing M9 supernatant, and transferring 50μL aliquots of worm pellet to 1.5mL tubes.

### Long-read sequencing:

DNA for long-read sequencing was extracted using the Promega Wizard^®^ HMW DNA Extraction Kit (cat. no. A2920), using the manufacturer protocol with minor modifications. Worm cuticles were broken by repeated freeze/thaw cycles where one tube of live worm pellet was placed at −80°C for five minutes, moved to 37°C until thawed, briefly vortexed, and then frozen again, for five cycles. All centrifuge steps were done at 4°C and alcohols were kept on ice until use. At the lysis step, an extra incubation step of 25 minutes at 65°C was added. PacBio sequencing was performed at the University of Miami’s John P. Hussman Institute for Human Genomics Sequencing Core Facility (RRID:SCR_017828).

#### Hi-C

Extra tubes of 50μL worm pellet were frozen at − 80°C using a Mr. Frosty^™^ freezing container (Thermo Scientific cat. no. 5100–001) to prevent ice crystal formation. Two tubes were then mailed on dry ice to Arima Genomics for High Coverage Chromatin Conformation Capture sequencing (Hi-C).

#### Genome Assembly

PacBio HiFi and Arima Hi-C libraries were assembled using Hifiasm v0.16.0 [6] using default parameters. BLAST v2.14.1 [4] was used to identify and remove contaminant contigs from the diploid and phased haploid assemblies. PacBio HiFi reads were mapped to the assembly using minimap2 v2.30 [20] with parameter -x map-hifi and read depth cutoffs were calculated with pbcstat from purge dups v1.2.6 [15]. Assembly self-alignment was performed with minimap2 v2.30 and parameters -xasm5 -DP to find duplications. Alterative haplotypes were subsequently removed with purge dups v1.2.6. QUAST v5.3.0 [16] with default parameters and BUSCO v6.0.0 [21] against lineage dataset Nematoda odb12 with option -m genome and --offline were used to quality check the assemblies between each step mentioned above.

#### Hi-C Mapping

Juicer v2.0 [9] was used for alignment and processing of Hi-C raw data using default parameters, with the --assembly option. YaHS v1.2.2 [32] was used for scaffolding. Juicebox v2.3.6 [25] was used for visualization and assessment.

### Gene and Repeat Annotation:

Gene annotation was performed with BRAKER3 v3.0.8 [12] on the softmasked assemblies using the protein dataset Nematoda odb10 and RNA sequence data downloaded from NCBI project PRJNA1256413 [22]. Prior to BRAKER3, genomes were softmasked with RepeatModeler2 [11] and RepeatMasker [29], and RNA reads were aligned to the genome with STAR v2.6.1a and option --outSAMstrandField intronMotif [8]. Briefly, BRAKER relies on 2 generalized hidden markov models for gene prediction, GeneMark (unsupervised) and AUGUSTUS (supervised) [3, 27]. The resulting protein sets are then combined by TSEBRA [13] to maximize BUSCO completeness scores. Protein predictions were then filtered for the longest isoform using AGAT v1.4.1 [7], specifically the scripts agat_sp_keep_longest_isoform.pl and agat_sp_extract_sequences.pl. Statistics were generated with agat_sp_statistics.pl. Functional annotations were done with InterproScan v5.68.100.0 with options -dp -goterms -pathways [17]. OrthoFinder v2.5.5 [10] was used to find single copy orthologs between JU4118 and *C. elegans*. The six major chromosomes were identified by location of single copy orthologs on *C. elegans* chromosomes. Nigon element classifications were assigned to single copy orthologs using a list of known gene:Nigon associations from [14].

Repetitive elements were annotated with EarlGrey v6.0.1 [1] and options -r nematoda -e yes. Briefly, EarlGrey employs a BLAST, Extract, Align, Trim (BEAT) process adapted from TEStainer (https://github.com/jamesdgalbraith/TEstrainer) along with subprocesses: Tandem Repeat Finder [2], MREPS [19], SA-SSR [24], LTR_FINDER [31], RepeatModeler2, and RepeatMasker. EarlGrey merges and defragments the annotations with RepeatCraft [30] to produce the final consensus library.

#### Snail Plots

Snail plots were generated by BlobTk v0.8.0 [5] using the soft-masked assemblies. Assembly BUSCO scores were recalculated for the snail plots using BUSCO v6.0.0 [21] and the nematoda_odb12 dataset.

## Figures and Tables

**Figure 1:**
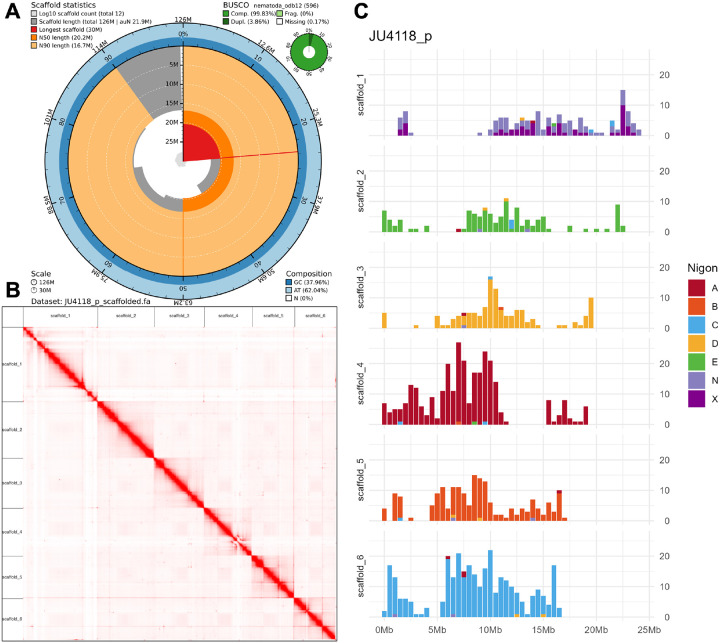
**A.** Snail plot (BlobTk) of assembly statistics for JU4118. The outer circumference of the plot represents the full length of the genome. The rings, from outer- to inner-most describe the features: the blue rings represent %GC content per scaffold; the pale orange ring represents N90 length in Mb; the bold orange ring represents N50 length in Mb; the red wedge is the longest scaffold length in Mb; the dark gray represents the length of each scaffold, arranged from longest to shortest in a clockwise direction; the light gray represents total scaffold count on a logarithmic scale. The top right circle describes BUSCO scores, displaying a summary of complete, duplicated, fragmented, and missing genes in the nematoda_odb12 set. **B.** Hi-C contact map of JU4118 assembly reveals the six chromosome-scale scaffolds (X sex chromosome, autosomes 1–5, ordered from largest to smallest. Scaffolds 7–12 are unplaced **C.** Scaffolds with single copy orthologs to *C. elegans* genes previously assigned to Nigon elements (A-E, N, X) in 100kb bins.

**Table 1.** Properties of *Caenorhabditis* species #65

Location found	Da Lat, Vietnam
Location coordinates	12.17523, 108.69884
Genome size (Mb)	126.4
Approximate coverage (x) – PacBio HiFi	170.9
Approximate coverage (x) – Illumina Hi-C	642.9
Number of reads (Gb) – PacBio HiFi	21.6
Number of reads (Gb) – Illumina Hi-C	81.3
GC content (%)	37.96
Number of protein coding genes	18,221
Repeats (%) – RepeatMasker	20.11
Repeats (%) – EarlGrey	24.6
SRA accession no. – PacBio HiFi	SRR38757051
SRA accession no. – Illumina Hi-C	SRR38757052
Isolated by	Marie-Anne Félix lab

## Data Availability

Bioinformatic scripts, workflows and software commands are available at https://github.com/jannafierst/HiC_Assemblies
